# The pequi pulp oil (*Caryocar brasiliense* Camb.) provides protection against aging-related anemia, inflammation and oxidative stress in Swiss mice, especially in females

**DOI:** 10.1590/1678-4685-GMB-2017-0218

**Published:** 2018-11-29

**Authors:** Mariana Matos Roll, Ana Luisa Miranda-Vilela, João Paulo Figueiró Longo, Tania da Silveira Agostini-Costa, Cesar Koppe Grisolia

**Affiliations:** ^1^ Departmento de Genética e Morfologia, Instituto de Ciências Biologicas, Universidade de Brasília, Brasilia, DF, Brazil; ^2^ Embrapa Recursos Genéticos e Biotecnologia (CENARGEN), Brasília, DF, Brazil

**Keywords:** aging-related inflammation and oxidative stress, comet assay, micronucleus, antioxidant, folk medicine

## Abstract

Continued exposure to reactive oxygen species and inflammation are the rationale behind aging theories and associated diseases. Scientific evidence corroborates the ethnomedicinal use of the oil of pequi (*Caryocar brasiliense* Camb.), a typical Brazilian Cerrado fruit, against oxidative damage to biomolecules and inflammation. We aimed to investigate *in vivo* the antioxidant and anti-inflammatory effects of pequi oil on hemogram and DNA damage in healthy young adult and older middle-aged Swiss mice of both genders. Animals, aged 6-7 and 11-12 months, were orally treated for 15 days with pequi oil at 30 mg/day. Blood samples were used for hemogram and comet assay, and bone marrow for micronucleus test. Female controls of 11-12 months had significantly lower haemoglobin and hematocrit than those of 6-7 months. Treatment with pequi oil improved this state, removing the differences. Pequi oil had no genotoxic or clastogenic effects and significantly increased lymphocytes and decreased neutrophils+monocytes in females of 11-12 months, removing the significant differences observed between controls of 6-7 and 11-12 months. The results suggest that dietary supplementation with pequi oil could protect against anemia, inflammation and oxidative stress related to aging, helping to prevent aging-related chronic degenerative diseases, mainly for females.

## Introduction

Aging is a physiological process characterized by a gradual decline in organ functions and by the development of age-related diseases arising from progressive oxidative damage to cellular structures such as DNA, proteins, and lipids (Gemma *et al.*, 2007; Papazzo *et al.*, 2010; Romano *et al.*, 2010). Change in an organism’s over time includes molecular events that may lead to cellular alterations that contribute, in turn, to organ senescence and system failure (Romano *et al.*, 2010).

Although different theories of aging exist, including evolutionary theories, the free radical theory, the mitochondrial theory, the immunologic theory and the inflammation theory (Romano *et al.*, 2010), the two most plausible, acceptable and durable ones are the free radical and mitochondrial theories (Gemma *et al.*, 2007; Romano *et al.*, 2010). The free-radical theory, proposed by Harman in the mid-1950s, suggests that reactive oxygen species (ROS) produced during aerobic respiration have deleterious effects on cell components and connective tissues, causing cumulative damage to membranes, proteins, and DNA over time and result in aging and degenerative diseases (Trifunovic, 2006, Gemma *et al.*, 2007) Developed from this, the mitochondrial theory, which Harman expanded in 1972, includes the involvement of mitochondria in the physiological processes of aging, since mitochondria are both producers and targets of ROS (Trifunovic, 2006). As damaged mitochondria progressively become less efficient, losing their functional integrity and releasing more ROS, the increased oxidative damage to the mitochondria culminates in an accumulation of dysfunctional mitochondria with age (Trifunovic, 2006, Gemma *et al.*, 2007). Because the byproducts of oxidative phosphorylation reactions can diffuse from mitochondria, reaching the nuclear DNA and inducing damage, this cellular process accounts for the background levels of oxidative damage to DNA detected in normal tissue (Miranda-Vilela *et al.*, 2010b).

Even though aerobic organisms are equipped with very efficient antioxidant defense mechanisms, and ROS and reactive nitrogen species (RNS) are important in maintaining homeostasis and cell survival by acting as cellular messengers in redox signaling (Gemma *et al.*, 2007; Yan, 2014), the oxidative damage to mitochondria over time can lead to a vicious cycle in which damaged mitochondria produce increased amounts of ROS, leading in turn to progressive increased damage (Trifunovic, 2006; Gemma *et al.*, 2007; Romano *et al.*, 2010). In fact, an increased number of dysfunctional mitochondria are found in a range of animal models and human tissues with increasing age (Trifunovic, 2006), and the ongoing DNA damage caused by ROS generated during oxidative metabolism is considered a key factor contributing to cell aging (Zhao *et al.*, 2007). So, continued exposure to ROS is the rationale behind the free radical and mitochondrial theories of aging (Papazzo *et al.*, 2010), and the progressive increase in cellular damage could be corrected (or at least reduced) by environmental, nutritional and pharmacological strategies (Romano *et al.*, 2010).

Within the above context, antioxidant supplementation is receiving growing attention (Fusco *et al.*, 2007). In this respect, strong scientific evidence corroborates the ethnomedicinal use of the oil of pequi (*Caryocar brasiliense* Camb.), a typical fruit of the Brazilian Cerrado that is rich in several natural antioxidants, to act against oxidative damage to biomolecules, inflammation (Miranda-Vilela *et al.*, 2008, 2009a,b,c, 2010a, 2011a), preneoplastic and neoplasic lesions (Miranda-Vilela *et al.*, 2011b, 2014; Palmeira *et al.*, 2015). Pequi is widely used by the local population in regional cookery and in folk medicine, where it is well known for its anti-inflammatory, tonic and aphrodisiac properties. The oil of its pulp has also been popularly used against bronchitis, colds, flu, and in tumor control (Almeida *et al.*, 2000, 2008; Mendes and Carlini, 2007). So, pequi oil could be considered an adaptogen, or in other words, a natural metabolic regulator that increases the ability of the organism to adapt to environmental factors and to avoid damage from such factors, such those that occur in oxidative stress and aging (Brekhman and Dardymov, 1969; Mendes and Carlini, 2007).

Aging-induced changes have been shown to adversely affect the quality of life in elderly populations (Papazzo *et al.*, 2010), and supporting evidence of antioxidant supplementation is still scarce and equivocal (Fusco *et al.*, 2007). By means of the above, and since pequi pulp is also rich in iron, we hypothesized that pequi oil could protect against age-related anemia, inflammation and oxidative stress. To test this hypothesis, we aimed to investigate the potential antioxidant and anti-inflammatory effects of pequi oil (*Caryocar brasiliense* Camb.) on hemogram and DNA damage (evaluated by comet assay and micronucleus test) in healthy young adult and older middle-aged Swiss mice of both genders. This choice was based on the following facts: (1) the young adult group is the reference for any age change, be it developmental, maturational, or senescent change (Flurkey *et al.*, 2007); (2) typically, the reproductive life is until about 7-8 months (Flurkey *et al.*, 2007; Jackson-Laboratory, 2007); (3) past this age, mice might exhibit some age-related change (e.g., female mice are retired from breeding at 8 months because litter size diminishes), although even mice as old as 12 months have been used as the “normal control” (Flurkey *et al.*, 2007); (3) a middle-aged group is often included in studies of aging to determine if an age-related change is progressive or is first expressed only in old age (Flurkey *et al.*, 2007); and (4) separating diseased animals from senescent animals can be confusing because both senescence and disease produce impairment, and because senescence processes increase the risk of age-related disease (Flurkey *et al.*, 2007). So, senescence is normal and will appear in all members of a species that live a normal life span, whereas the vast majority of diseases is abnormal and appear only in a subset of the species (Flurkey *et al.*, 2007). Hence, we choose to use older middle-aged mice rather than old mice to avoid any bias arising from age-related diseases (age-related disease can skew results) (Flurkey *et al.*, 2007).

## Materials and Methods

### Chemicals

The anesthetics ketamin and xylazin were obtained as chloridrate. Ketamin, sold as Dopalen 100 mg/mL, was obtained from Ceva Animal Health Ltd (São Paulo, Brazil), and xylazin (Coopazine 20mg/mL) came from Coopers (São Paulo, Brazil). Butylated hydroxytoluene (BHT) was purchased from Merck (Brazil); ascorbic acid (Citroplex), from Neo Química Laboratory (Goiás, Brazil); and 1,1-Diphenyl-2-picryl-hydrazyl (DPPH from Sigma-Aldrich (Brazil).

### Plant material and chemical analyses

Pequi oil was extracted from the pulp of fresh pequi through mechanical pressure and centrifugation to obtain extra-virgin oil. The oil was vacuum-filtered and stored in amber bottles under refrigeration. The relative fatty acid and carotenoid compositions of the pequi pulp oil have been previously reported (Miranda-Vilela *et al.*, 2009b, 2011b, 2013), but since variations in the composition of the pequi’s carotenoids occur as a function of the state of ripeness at the time of harvesting (Oliveira *et al.*, 2006), its total carotenoid content was analyzed using the methodology described by Higby (1962). Pequi oil macronutrients and micronutrients were also quantified by the Laboratory of Agrochemistry and Environment of the State University of Maringá (UEM, Brazil) using atomic absorption spectrometry (K, Ca, Mg, Cu, Fe, Mn and Zn) or UV-Vis spectrometry (P).

Total phenolic content was measured using the Folin-Ciocalteu method as described by Swain and Hillis (1959). A calibration curve was constructed from the absorbance of increasing concentrations (ranging from 0.02 to 0.14 mg/mL) of gallic acid (phenolic acid was used as standard) at 760 nm. The pequi oil was diluted in ethanol P.A. and, after the reaction of the diluted samples with the Folin-Ciocalteou reagent in alkaline medium, the reading was performed in a spectrophotometer at 760 nm. The content of total phenolic compounds was calculated from the equation of the line obtained in the calibration curve of gallic acid, where the results, determined from the regression equation of the calibration curve (y= 0.0008x – 0.0198; R^2^= 0.998), were expressed as mg gallic acid equivalents (GAE) per gram of the sample.

The antioxidant activity of the pequi oil was evaluated by the 2,2-diphenyl-1-picryl-hydrazyl-hydrate (DPPH) free radical scavenging method (DPPH assay), according to Brand-Williams *et al.* (1995), with adaptations from Razali *et al.* (2008) and Atmani *et al.* (2009), using methanol as solvent. Due to the immiscibility of the pequi oil in the solvent, the suspensions were stirred and then centrifuged at 78.4 x *g* for 10 seconds to separate the phases, where only the supernatant, soluble in methanol, was collected for interaction with DPPH. The concentration of the DPPH solution in methanol was of 0.04 mg/mL. For the reaction, 900 μL of DPPH solution and 100 μL of the pequi oil were used, or for the antioxidant controls butylated hydroxytoluene (BHT) and ascorbic acid, diluted in methanol at concentrations of 0.5 to 500 mg/mL. The mixtures reacted for 20 minutes in a place protected from light. The absorbances were measured in a spectrophotometer (Molecular Devices Spectramax M2) at a wavelength of 517 nm, and analyses were performed in triplicate. Results were expressed as percentage inhibition of DPPH (% inhibition), using the following formula:

$$\%\;\text{inhibition}\;=\frac{\left[\text{A}0-(\text{A}1-\text{AS})\right]}{\text{A}0}\times 100$$

Where A0 is absorbance of DPPH, A1 is absorbance of DPPH + pequi oil (or controls), AS is pequi oil absorbance (or controls)

Values of % inhibition were used to calculate EC50 (minimum concentration to reduce the DPPH free radical by 50%) by non-linear regression.

A voucher of the pequi specimen (*Caryocar brasiliense* Camb.) was deposited in the herbarium of the University of Brasilia (UnB) by Professor Cassia Munhoz (PhD), collection number 7402, registration number 165.857.

### Animals, treatments and experimental design

Female and male Swiss mice were purchased from the Multidisciplinary Center for Biological Investigation in Laboratory Animal Science (Cemib) of the State University of Campinas (Unicamp, SP, Brazil), housed in plastic cages with ventilation and air circulation under standard conditions of 12 hours dark/light cycle, controlled temperature (20 °C ± 2 °C) and free access to food and water. The animals were maintained in the animal facility of the Department of Genetics and Morphology of the University of Brasilia (UnB). They were randomly distributed in four treatment groups (6 per cage), aged 6 to 7 months, initially weighing 38.10 ± 4.34 g (females), and 39.66 ± 1.68 g (males); and aged 11 to 12 months, initially weighing 48.83 ± 4.71 g (females) and 37.67 ± 1.75 g (males). Pequi oil was administered for 15 days at 30 mg/animal/day, according to previously reported tests (Miranda-Vilela *et al.*, 2008, 2011b, 2014), where the daily dose of the antioxidants was calculated using the dose translation formula developed by Reagan-Shaw *et al.* (2007), obeying the maximum daily dose of provitamin A carotenoids (25 mg) for adult humans recommended by the National Agency for Sanitary Surveillance (ANVISA). Negative controls (NC) received 30 mg/animal/day of filtered water orally administered.

At the end of the experiment, all mice were euthanized by cervical dislocation after anesthesia, according to the guidelines on Euthanasia of the Federal Council of Veterinary Medicine (CFMV) (Conselho Federal de Medicina Veterinária, 2013). Before euthanasia, the animals were anesthetized by an intraperitoneal administration of ketamine (80 mg/kg) and xylazin (10 mg/kg) in a final dose of 0.1 mL/30 g. Blood samples (1 mL) collected by cardiac puncture were used to carry out the hemogram in a multiple automated hematology analyzer for veterinary use (Sysmex pocH-100iV Diff. Curitiba/Paraná, Brazil), and the comet assay (alkali method), carried out by a standard method proposed by Singh *et al.* (1988) with modifications (Miranda-Vilela *et al.*, 2008, 2011b, 2014). In short, 20 μL total blood of each animal was mixed with 120 μL of 0.5% low-melting-point agarose (LMA) in phosphate-buffered saline (PBS) at 37 °C and pipetted onto eight microscope slides pre-coated with a layer of 1.5% normal-melting-point agarose prepared in PBS. Slides were then immersed in a freshly prepared cold (4 °C) lysis solution (2.5 M NaCl, 100 mM Na_2_EDTA, 10 mM Tris, pH 10.0-10.5, 1% lauroyl sarcosine, with 1% Triton X-100 and 10% dimethyl sulfoxide added fresh) for 1 h at 4 °C. After lysis, slides were placed in a horizontal gel electrophoresis tank with fresh alkaline electrophoresis buffer (300 mM NaOH, 1 mM Na_2_EDTA, pH > 13.0), left in the solution for 40 min at 4 °C, and then proceeding to electrophoresis at 4 °C for 30 min at 25 V and 300 mA. Subsequently, slides were immersed three times (3 x 5 min), in neutralizing solution (0.4 M Tris, pH 7.5), fixed for 5 min in 100% ethanol, stained with EtBr (20 μg/ml) and analyzed with a Zeiss Axioskop 2 Fluorescence Microscope (filter 510-560 nm, barrier filter 590 nm) with a total magnification of 400x. One hundred comets on each slide were scored visually by a trained professional as belonging to classes 0-4, as proposed by Collins *et al.* (1995), and the DNA damage was calculated according to Jaloszynski *et al.* (1997), giving a maximum possible score of 400, corresponding to 100 cells in class 4. For each animal, the slides were prepared in duplicate.

After euthanasia, bone marrow cells were surgically removed to carry out the micronucleus (MN) test according to a standard method (Schmid, 1975) with modifications (Estevanato *et al.*, 2011). Briefly, the bone marrow cells were collected with 1 mL of fetal bovine serum (FBS) and centrifuged at 160 x *g* for 5 min. Supernatant was discarded and 50 μL of FBS was added and homogenized to the pellet. Afterward, the suspension was smeared on glass slides (the slides were prepared in duplicate), fixed by methanol for 10 minutes, and stained with Giemsa dye. A total of two thousand erythrocytes were counted per mouse (1000 normochromatic erythrocytes, NCE, and 1000 polychromatic erythrocytes, PCE) in light microscopy (1000 X magnification), using a Zeiss Axioskop 2 microscope, and the frequency of micronuclei (MN) in PCE and NCE, and the percentage of polychromatic erythrocytes (%PCE) were calculated.

The study was performed according to the international, national and institutional rules considering animal experiments, clinical studies and biodiversity rights. It was approved by the institutional Ethics Committee for Animal Research (Institute of Biological Science, University of Brasília), UnBDoc number 5330/2011, and authorized by the Ministry of the Environment as required under the framework of the United Nations Convention on Biodiversity.

### Statistical analyses

Statistical analysis was carried out using SPSS (Statistical Package for the Social Sciences) version 17.0, Data were expressed as mean ± SEM (standard error of mean) and values of *p* < 0.05 were considered statistically significant. The continuous variables were tested for normal distribution with the Shapiro-Wilk test. Differences among the groups were investigated by ANOVA or Kruskal-Wallis tests, depending on whether the data were normally distributed or not, followed respectively by Bonferroni or Mann-Whitney U tests.

## Results

### Chemical analyses of the pequi oil

The relative composition of pequi (*Caryocar brasiliense* Camb.) pulp oil is shown in Table 1 (Ramos *et al.*, 2001; Azevedo-Meleiro and Rodriguez-Amaya, 2004; Oliveira *et al.*, 2006; Lima *et al.*, 2007; Miranda-Vilela *et al.*, 2009b). The inhibition percentage of the free radical pre-formed by the pequi oil and controls allowed the calculation of the EC50 (concentration required to reduce 50% of DPPH) (Table 2).

**Table 1:** Relative composition of pequi (*Caryocar brasiliense* Camb.) pulp oil.

Fatty acids* ^,(1)^						Carotenoids		
(%)						(mg/100 g of pequi pulp fruit)		
Saturated		Monounsaturated		Polyunsaturated		Provitamin A^(2-5)^	Lycopene (2 [Table-fn tfn4] [Table-fn tfn5]-5)	Total*
Palmitic	41.78	Oleic	54.28	Linoleic	1.36	6.26−11.5	1.12−2.08	27.75 ± 0.11
Stearic	1.28	Palmitoleic	0.67	Linolenic	0.51			
Arachidic	0.12							
Total phenol content*	Macronutrients*				Micronutrients*			
(mg.GAE.g^-1^)	(g/kg)				(mg/kg)			
nd	Mg	Ca	K	P	Fe	Cu	Mn	Zn
	0.114	0.97	0.042	nd	186.8	nd	2.02	2.03

^*^ present study;

^1^ Miranda-Vilela *et al.*, 2009b;

^2^ Azevedo-Meleiro and Rodriguez-Amaya, 2004;

^3^ Lima *et al.*, 2007;

^4^ Oliveira *et al.*, 2006;

^5^ Ramos *et al.*, 2001. nd= not detected. Data of total carotenoids are expressed as mean ± standard deviation (SD) of three absorbance readings.

**Table 2:** Antioxidant activity of the pequi pulp oil by the DPPH assay. As controls, the antioxidants butylated hydroxytoluene (BHT) and ascorbic acid were used.

Samples	EC_50_ (mg/mL)	95% CI
BHT	0.0006	0.0056 - 0.00652
Ascorbic acid	0.012	0.0103 - 0.0145
Pequi oil	26.26	21.99 - 31.36

95% CI= 95% confidence intervals; BHT= Butylated hydroxytoluene.

### Hematological evaluations

#### *Erythrogram*

Between genders there were significant differences for controls of the age group of 6-7 months in the values of red cell distribution width (RDW) and of the age group of 11-12 months in the values of hemoglobin (HGB), which were higher in males. On the other hand, males of 6-7 months treated with pequi oil had significantly lower values of HGB and hematocrit (HCT) and higher values of mean corpuscular volume (MCV) than females.

Female controls of 11-12 months had significantly decreased HGB and HCT compared to controls of 6-7 months. The treatment with pequi oil improved this state, removing such significant differences (Table 3).

**Table 3:** Results of erythrogram of elderly female (F) and male (M) Swiss albino mice after treatment with pequi oil (30 mg/animal/day) administered orally for 15 consecutive days. Negative controls (NC) received filtered water administered in the same way.

Group	Treatments/age groups	RBC	HGB	HCT	MCV	MCH	MCHC	RDW
		(x 10^6^/μL)	(g/dL)	(%)	(fL)	(pg)	(g/dL)	(%)
**1**	NC (F) 6-7 months	8.23 ± 0.44	12.34 ± 0.66	30.34 ± 1.49	36.86 ± 0.40	14.98 ± 0.50	40.66 ± 1.06	13.18 ± 0.69
**2**	PO (F) 6-7 months	8.74 ± 0.30	12.78 ± 0.38	32.17 ± 1.11^*a^	36.80 ± 0.36	14.62 ± 0.26	39.77 ± 0.56	12.75 ± 0.57
**3**	NC (F) 11-12 months	6.95 ± 1.61	10.07 ± 2.19^*a^	25.38 ± 4.97^*a^	37.00 ± 2.56	14.55 ± 0.69	39.45 ± 1.29	15.75 ± 3.10
**4**	PO (F) 11-12 months	7.84 ± 1.22	10.77 ± 1.52^**b^	27.57 ± 4.00^**b^	35.22 ± 0.58^**b^	13.77 ± 0.28^*b^	39.08 ± 0.50	14.13 ± 1.04
	*p*-values	0.056	0.012	0.014	0.018	0.003	0.059	0.036
**5**	NC (M) 6-7 months	7.34 ± 1.12	11.07 ± 1.49	28.10 ± 3.30	38.52 ± 2.13	15.15 ± 0.74	39.35 ± 0.79	18.37 ± 2.67#
**6**	PO (M) 6-7 months	7.57 ± 0.39	11.62 ± 0.72#	28.80 ± 1.46#	38.03 ± 0.65#	15.33 ± 0.43	40.33 ± 0.84	16.7 ± 1.73#
**7**	NC (M) 11-12 months	7.89 ± 0.57	11.80 ± 0.82#	29.47 ± 2.29	37.36 ± 1.10	14.96 ± 0.47	40.09 ± 0.94	15.86 ± 1.19
**8**	PO (M) 11-12 months	7.45 ± 0.99	11.07 ± 1.33	27.15 ± 3.05	37.02 ± 1.10#	15.07 ± 0.35#	40.72 ± 0.82	16.00 ± 1.60
	*p*-values	0.641	0.662	0.445	0.237	0.620	0.069	0.097
	*p*-values (Total)	0.069	0.004	0.003	0.005	**0.000**	**0.017**	**0.000**

Data correspond to mean and standard error of mean (SEM). NC= negative control; PO= treatment with pequi oil at 30 mg/animal/day (or ~1 g/kg body weight); RBC= Red Blood Cells; HGB= Hemoglobin; HCT= Hematocrit; MCV= Mean Corpuscular Volume; MCH= Mean Corpuscular hemoglobin; MCHC= Mean corpuscular hemoglobin concentration; RDW = Red cell distribution width (represents an indication of the amount of variation − anisocytosis − in cell size); g/dL= grams per deciliter; fL= femtoliters; pg= picograms. P-values highlighted in bold were generated by ANOVA, while other *p*-values were generated by the Kruskal-Wallis test. The symbol # indicates significant differences between female and male mice in the same treatments, while the superscript letters indicate significant differences in the 2-to-2 comparisons, detected by the Bonferroni or the Mann-Whitney U tests within each gender, with a = significant compared to group 1; b = significant compared to group 2. Asterisks indicate significant differences at

^*^ *p*< 0.05

^**^ *p* < 0.01).

#### *Leukogram*

For the controls, significant differences between genders were found for both age groups in lymphocytes (%) and neutrophils+monocytes (%), where males showed lower values for lymphocytes (%) and higher values for neutrophils+monocytes (%) than females. Pequi oil removed these differences in the age group of 11-12 months.

For females, controls of 11-12 months had significantly diminished lymphocytes (%) and increased neutrophils+monocytes (%) compared to those of 6-7 months. The treatment with pequi oil improved this situation, removing the significant differences with respect to the control. For this gender, pequi oil also resulted in significantly decreased eosinophils (%) in comparison with controls in the age group of 6-7 months.

For males, the treatment with pequi oil decreased the total white blood cells (WBC) count, and this was significant for the age group of 6-7 months. For the age group of 11-12 months, there were significant differences in the percentage of lymphocytes and neutrophils+monocytes between control males and those males treated with pequi oil, where pequi oil significantly increased lymphocytes (%) and decreased neutrophils+monocytes (%) (Table 4).

**Table 4:** Results of leukogram of elderly female (F) and male (M) Swiss albino mice after treatment with pequi oil (30 mg/animal/day) administered orally for 15 consecutive days. Negative controls (NC) received filtered water administered in the same way.

Group	Treatments/age groups	WBC (x10^3^/μL)	Lymphocytes (%)	Neutrophils + Monocytes (%)	Eosinophils (%)
1	NC (F) 6-7 months	4.02 ± 1.70	77.22 ± 2.72	21.10 ± 2.23	1.68 ± 1.32
2	PO (F) 6-7 months	4.13 ± 0.65	76.03 ± 4.92	23.48 ± 4.40	0.48 ± 0.84^*^ ^a^
3	NC (F) 11-12 months	9.02 ± 12.45	47.23 ± 17.33^**^ ^a^	49.92 ± 16.28^**^ ^a^	1.37 ± 1.37
4	PO (F) 11-12 months	3.40 ± 0.98	58.72 ± 7.16^*^ ^b^	39.52 ± 6.26^*^ ^b^	1.77 ± 3.25
	*p*-values	0.598	**0.000**	**0.000**	0.083
5	NC (M) 6-7 months	12.15 ± 4.92	29.27 ± 8.21#	69.47 ± 7.09#	1.27 ± 1.34
6	PO (M) 6-7 months	7.33 ± 2.45^*^ ^a^	43.43 ± 8.30#	55.20 ± 9.48#	1.37 ± 1.37
7	NC (M) 11-12 months	12.47 ± 6.70	19.21 ± 8.65#	80.06 ± 8.37#	0.73 ± 0.71
8	PO (M) 11-12 months	6.00 ± 3.22	61.53 ± 19.76^**^ ^c^	37.03 ± 20.27^**^ ^c^	1.43 ± 0.65
	*p*-values	**0.019**	**0.000**	**0.000**	0.429
	*p*-values (Total)	**0.001**	**0.000**	**0.000**	0.101

Data correspond to mean and standard error of mean (SEM). NC= negative control; PO= treatment with pequi oil at 30 mg/animal/day (or ~1 g/kg body weight); WBC = Total White Blood Cells. P-values highlighted in bold were generated by ANOVA, while other *p*-values were generated by the Kruskal-Wallis test. The symbol # indicates significant differences between female and male mice in the same treatments, while the superscript letters indicate significant differences in the 2-to-2 comparisons, detected by the Bonferroni or the Mann-Whitney U tests within each gender, with a= significant compared to group 1; b= significant compared to group 2; c= significant compared to group 7. Asterisks indicate significant differences at

^*^ *p* < 0.05

^**^ *p* < 0.01).

In Table 4 it is possible to observe that there is a significant difference in the percentage of lymphocytes and neutrophils + monocytes between control males (11-12) and males (11-12) treated with pequi oil.

#### *Plateletgram*

Significant differences between genders appeared only for the mean platelet volume (MPV) after treatment with pequi oil in the age group of 6-7 months. For females, values of MPV and platelet large cell ratio (P-LCR) were significantly higher in the age group of 11-12 months compared to the age group of 6-7 months after pequi oil supplementation. For males, the only significant difference was related to the platelet distribution width (PDW), where the pequi oil significantly reduced this value in the age group of 11-12 months with respect to the control and also compared to the age group of 6-7 months treated with pequi oil (Table 5).

**Table 5:** Results of plateletgram of elderly female (F) and male (M) Swiss albino mice after treatment with pequi oil (30 mg/animal/day) administered orally for 15 consecutive days. Negative controls (NC) received filtered water administered in the same way.

Group	Treatments/age groups	PLT	MPV	P-LCR	PDW
		(x 10^3^/μL)	(fL)	(%)	(fl)
1	NC (F) 6-7 months	1082.60 ± 168.84	6.77 ± 0.76	10.07 ± 3.67	6.90 ± 0.66
2	PO (F) 6-7 months	1288.00 ± 77.28	6.23 ± 0.29	6.68 ± 1.54	6.62 ± 0.26
3	NC (F) 11-12 months	1044.17 ± 504.32	6.70 ± 0.30	8.28 ± 1.66	6.98 ± 0.18
4	PO (F) 11-12 months	1133.67 ± 393.22	6.72 ± 0.23 ^*^ ^a^	10.54 ± 2.57 ^*^ ^a^	6.54 ± 0.25
	p-values	0.630	0.120	0.057	0.148
5	NC (M) 6-7 months	1793.67 ± 462.53	7.10 ± 0.35	10.27 ± 3.83	7.30 ± 0.11
6	PO (M) 6-7 months	1792.67 ± 495.07	6.70 ± 0.27#	7.82 ± 1.80	7.13 ± 0.38
7	NC (M) 11-12 months	1657.29 ± 344.39	6.93 ± 0.35	9.77 ± 2.48	7.19 ± 0.41
8	PO (M) 11-12 months	1582.83 ± 727.89	6.68 ± 0.40	9.62 ± 4.73	6.55 ± 0.23^*^ ^b^.^**^ ^c^
	*p*-values	0.863	0.202	0.465	**0.002**
	*p*-values (Total)	**0.011**	**0.023**	0.150	**0.000**

Data correspond to mean and standard error of mean (SEM). NC= negative control; PO= treatment with pequi oil at 30 mg/animal/day (or ~1 g/kg body weight); PLT= platelet count; MPV= mean platelet volume; P-LCR= platelet large cell ratio; PDW= platelet distribution width; fl= femtoliters. P-values highlighted in bold were generated by ANOVA, while other *p*-values were generated by the Kruskal-Wallis test. The symbol # indicates significant differences between female and male mice in the same treatments, while the superscript letters indicate significant differences in the 2-to-2 comparisons, detected by the Bonferroni or the Mann-Whitney U tests within each gender, with a= significant compared to group 2; b= significant compared to group 6; c= significant compared to group 7. Asterisks indicate significant differences at

^*^ *p* < 0.05

^**^ *p* < 0.01).

### Genotoxicity evaluations by Comet assay and Micronucleus (MN) test

For the control group, significant differences between genders were showed in the comet assay in the age group of 11-12 months, where males had lower levels of total DNA damage than females. For the treatment with pequi oil, these differences appeared only in the age group of 6-7 months (Table 6).

**Table 6:** Results of Comet assay (total DNA damage and the correspondent % total damage) obtained from the peripheral blood samples of elderly female (F) and male (M) Swiss albino mice after treatment with pequi oil (30 mg/animal/day) administered orally for 15 consecutive days. Negative controls (NC) received filtered water administered in the same way.

Group	Treatments/age groups	Total DNA damage (au)	%Total damage
**1**	NC (F) 6-7 months	155.00 ± 11.34	38.75 ± 5.67
**2**	PO (F) 6-7 months	142.42 ± 4.73	35.60 ± 2.90
**3**	NC (F) 11-12 months	165.67 ± 8.10	41.42 ± 4.96
**4**	PO (F) 11-12 months	164.00 ± 9.74	41.00 ± 5.96
	*p*-values	0.243	
**5**	NC (M) 6-7 months	182.75 ± 6.18	45.69 ± 3.09
**6**	PO (M) 6-7 months	183.40 ± 7.80#	45.85 ± 4.36#
**7**	NC (M) 11-12 months	142.33 ± 3.18#	35.58 ± 1.95#
**8**	PO (M) 11-12 months	140.83 ± 3.83	35.21 ± 2.35
	*p*-values	**0.000**	
	*p*-values (Total)	**0.005**	

The data correspond to the means ± standard error of the mean (SEM). au= arbitrary units; NC= negative control; PO= treatment with pequi oil at 30 mg/animal/day (or ~1 g/kg body weight). *P*-values highlighted in bold were generated by ANOVA, while other *p*-values were generated by the Kruskal-Wallis test. The symbol # indicates significant differences between female and male mice in the same treatments.

The MN test showed no clastogenic effect of the pequi oil, although there was a significant difference between the age groups of females in the cellular proliferation index (%PCE), where animals of 11-12 months had lower values than those of 6-7 months. Treated males of 6-7 months presented higher %PCE values than male controls of the same age. The control group for animals aged 6-7 months, in turn, showed a significant difference between genders regarding this parameter, where males had lower values (Table 7).

**Table 7:** Frequencies of micronucleus (MN) evaluation and polychromatic erythrocytes (% PCE) of bone marrow cells of elderly female (F) and male (M) Swiss albino mice after treatment with pequi oil (30 mg/animal/day) administered orally for 15 consecutive days. Negative controls (NC) received filtered water administered in the same way.

Group	Treatments/age groups	MN-NCE	Polychromatic erythrocytes (PCE)	
			MN-PCE	Cellular proliferation index (%PCE)
1	NC (F) 6-7 months	0.40 ± 0.55	1.60 ± 1.52	49.63 ± 4.77
2	PO (F) 6-7 months	0.17 ± 0.41	0.33 ± 0.82	55.52 ± 17.11
3	NC (F) 11-12 months	0.33 ± 0.52	0.50 ± 0.84	46.17 ± 4.33
4	PO (F) 11-12 months	0.17 ± 0.41	0.17 ± 0.41	42.18 ± 4.78^*^ ^a^
	*p*-values	0.761	0.106	0.039
5	NC (M) 6-7 months	1.17 ± 1.17	1.00 ± 0.89	40.80 ± 4.97#
6	PO (M) 6-7 months	0.20 ± 0.45	0.60 ± 0.55	50.51 ± 5.23^*^ ^b^
7	NC (M) 11-12 months	0.50 ± 0.84	0.50 ± 0.55	42.10 ± 5.03
8	PO (M) 11-12 months	0.33 ± 0.52	1.50 ± 1.87	49.83 ± 8.84
	*p*-values	0.107	0.431	**0.030**
	*p*-values (Total)	0.495	0.222	**0.014**

Data correspond to mean and standard error of mean (SEM). MN-NCE and MN-PCE= micronucleus frequency for normochromatic erythrocytes (NCE) and polychromatic erythrocytes (PCE), respectively. NC= negative control; PO= treatment with pequi oil at 30 mg/animal/day (or ~1 g/Kg body weight). *P*-values highlighted in bold were generated by ANOVA, while other *p*-values were generated by the Kruskal-Wallis test. The symbol # indicates significant differences between female and male mice in the same treatments, while the superscript letters indicate significant differences in the 2-to-2 comparisons, detected by the Bonferroni or the Mann-Whitney U tests within each gender, with a= significant compared to group 2; b= significant compared to group 5. Asterisks indicate significant differences at

^*^ *p* < 0.05

^**^ *p* < 0.01).

## Discussion

Among the several *in vitro* methods used to evaluate the antioxidant activity of samples of interest, the DPPH method is faster, simpler and less expensive than other test models (Alam *et al.*, 2013). In this respect, the inhibition percentage of the free radical pre-formed by the pequi oil and controls allowed the calculation of the EC50 (concentration required to reduce 50% of DPPH), where the pequi oil showed an antioxidant potential in relation to DPPH radical, but, as expected, with lesser activity than the controls, because of the immiscibility of the pequi oil in the solvent and the necessity of separating and collecting the soluble phase in methanol to perform the DPPH assay.

However, the antioxidant activity of samples of interest should not be concluded based on a single antioxidant test model, although in practice it is difficult to compare fully one method to other one (Alam *et al.*, 2013). In this sense, even if no total phenol content was detected by the Folin-Ciocalteu method, a method which detects all classes of water-soluble polyhydroxyphenolic compounds (Turkmen *et al.*, 2006; Rocha *et al.*, 2011) pequi oil was diluted in ethanol P.A., where it is poorly soluble. As the content of total phenolic compounds in the pequi pulp has been reported as higher than that found in most fruits consumed in Brazil (Lima *et al.*, 2007), although the organic solvent systems with different polarities included absolute methanol and ethanol that were used in these two methods, our results are in accordance with the observation that in the polyphenol extraction, a single extraction compared to multiple extraction procedure is not sufficient (Turkmen *et al.*, 2006). Also, it can be concluded that it is not clear which solvent system is more effective for evaluating antioxidant activity and extracting total phenolics, mainly because pequi oil is soluble only in non-polar solvents, such as chloroform and n-hexane (Miranda-Vilela *et al.*, 2009b). On the other hand, pequi oil is rich in several other natural antioxidants, including vitamin E (Cardoso *et al.*, 2013) and carotenoids (Ramos *et al.*, 2001; Azevedo-Meleiro and Rodriguez-Amaya, 2004; Oliveira *et al.*, 2006; Lima *et al.*, 2007).

Generally referred to as vitamin E are the tocols (α, β, δ, γ tocopherol) and tocotrienols (α, β, δ, γ tocotrienol). They are well known for their established health benefits, including antioxidant, neuroprotective, and anti-inflammatory properties, where α-tocopherol is the most potent among them, because it is able to inhibit the lipoperoxidation chain reaction, preventing even the oxidation of low density lipoproteins (LDL) (Cerqueira *et al.*, 2007; Singh *et al.*, 2013). As for carotenoids, these are important in protecting cells and organisms against oxidative damage (Uddin and Ahmad, 1995). The carotenoids that have been most studied in this regard are β-carotene, lycopene, lutein, and zeaxanthin (Johnson, 2002), all of them present in pequi pulp oil (Ramos *et al.*, 2001; Azevedo-Meleiro and Rodriguez-Amaya, 2004; Oliveira *et al.*, 2006). Due to their highly conjugated double-bond system, carotenoids are extremely efficient quenchers of singlet oxygen, and it has been proposed that dietary β-carotene in combination with other antioxidants protects against lipoprotein oxidation and thus may play a potentially important role in retarding the progression of atherosclerosis (Uddin and Ahmad, 1995). So, dietary carotenoids are thought to provide health benefits by decreasing the risk of disease, particularly certain cancers and eye disease (Johnson, 2002). Thus, in the context of the above, the use of the *in vivo* effects of pequi pulp is justified and will be discussed further below.

Physiologic changes over the years of a long life seem to be responsible for the impairment of regulation or function of many organ systems (Kelso, 1990). In view of the facts that: (1) function is often measured in clinical medicine by laboratory testing and, for the most part, the laboratory values obtained in elderly subjects seem to fall into our traditional or so-called normal ranges, so little evidence supports the need for separate sets of reference ranges for the elderly (Kelso, 1990); and (2) the mouse is the animal model most widely used to study the pathogenesis and treatment of human diseases (Mazzaccara *et al.*, 2008), we considered the hematological reference values for younger adults to compare with our values. This is because mice as old as 12 months have also been used as the “normal control” (Flurkey *et al.*, 2007), and it has been reported that when analyzing hematological data from mice, not only should controls be used as the primary comparison for the interpretation of treatment-related changes, but also comparisons should be done with reference intervals to put hematologic changes in perspective (Everds, 2007). However, general significant differences in hematological data have been observed not only among the genders, but also among the ages of Swiss mice at 30, 45, 60, 75, 90, 105 and 120 days after birth (Restell *et al.*, 2014).

Also, only one article related to aging and reference intervals of the main hematological parameters was found, and it reported significant differences between ages for RBC, HGB, HCT, WBC, and PLT during the 1-year life span (Mazzaccara *et al.*, 2008). Although this work was performed with inbred mice, and our work was with outbred mice, our results corroborate the former study, at least regarding the female controls of 6-7 months and 11-12 months in the HGB, HCT and WBC parameters, the latter mainly related to lymphocytes and neutrophils+monocytes. Our work also corroborates the previous suggestion that therapeutic intervention should be evaluated against gender- and age-dependent reference intervals (Mazzaccara *et al.*, 2008), as discussed below.

The hematology of the mouse has been studied extensively (Everds, 2007), where RBC, HGB and HCT values have been reported as being higher in females than males (Mazzaccara *et al.*, 2008; Restell *et al.*, 2014; Araújo *et al.*, 2015), corroborating our results. Moreover, deformability is the characteristic that allows the normal erythrocyte to circulate through capillaries. This phenomenon depends on cell geometry, internal viscosity and viscoelastic properties of the erythrocyte membrane (Patavino *et al.*, 2006). Aged mouse erythrocytes have decreased deformability due to diminished surface area and cellular dehydration; osmotic fragility and the number of higher density erythrocytes also increase with age (Abe *et al.*, 1984; Everds, 2007). Thus, they are more fragile, less deformable, and more susceptible to shear stress and oxidative damage. Although anemia should not be accepted as an inevitable consequence of aging, it is a common issue in the elderly and its prevalence increases with age, being found in approximately 80 percent of elderly patients; the most common causes are related to chronic disease and iron deficiency (Smith, 2000). Some authors suggest that in iron deficiency anemia there is a reduction in the survival of the microcytic and hypochromic RBCs, and that one of the main factors would be their reduced flexibility. This fact would be related to the lower internal hemoglobin content and unfavorable surface/volume ratio of the cell, with consequent early sequestration by the reticuloendothelial system (Patavino *et al.*, 2006). As pequi is rich in iron, this could explain the increase in HGB and HCT and, consequently, the lack of significant differences observed for HGB and HCT in female controls of 11-12 months compared to 6-7 months. Furthermore, estrogens have antioxidant properties that are due to their ability to bind to estrogen receptors and to up-regulate the expression of antioxidant enzymes via intracellular signaling pathways. Mitochondria, where the estrogen receptors have been identified, are key organelles in the development of age-associated cellular damage. Estradiol prevents the onset of the mitochondrial pathway of apoptosis by a direct effect on the organelle that stopsthe formation of reactive oxygen species (ROS) by mitochondria in a saturable manner, protecting mitochondrial integrity, and preventing the apoptogenic leakage of cytochrome c from mitochondria (and as a result the mitochondrial content of cytochrome c is maintained high)(Borrás *et al.*, 2010). Since we worked with young adult and older middle-aged mice of both genders, the pequi oil, due to its antioxidant activity, possibly could be favoring this antioxidant effect of estrogen in females.

A decreased antioxidant capacity is associated with aging, causing overwhelming stress on physiological functions. This process increases damage to DNA, proteins and lipids (Papazzo *et al.*, 2010), favoring inflammation, and aging has also been reported as resulting in chronic low-grade inflammation that is associated with increased risk for disease, poor physical functioning and mortality (Woods *et al.*, 2012). Although we did not use classic inflammation markers or cytokine dosages, our results corroborate this, as do the previously reported anti-inflammatory properties of pequi oil (Miranda-Vilela *et al.*, 2009c). Indeed, the existence of a difference between genders found in our study corroborates the literature, in which there are reports of a significant effect for animal age and interaction between gender and age, where the increase in age also implied a higher number of leukocytes for males; the percentage of neutrophils also had a significant effect according to gender and age (Restell *et al.*, 2014).

In mice, the most common leukocyte in the peripheral blood is the lymphocyte (approximately three-fourths of the WBC) followed by neutrophils; small changes in the number of neutrophils may be biologically significant and reflected in the total leukocyte count (Everds, 2007). Neutrophils have been demonstrated not only as major effectors of acute inflammation, but also to contribute to chronic inflammatory conditions and adaptive immune responses, besides facilitating recruitment of monocytes into inflamed tissue sites (Kolaczkowska and Kubes, 2013). Moreover, in both humans and experimental animals, aging leads to a decrease in lymphocyte-dependent immunity (humoral and cellular immune responses) (Ahmad *et al.*, 2009). So, the effects of pequi oil on increasing lymphocytes (%) and decreasing neutrophils+monocytes (%) in the age group of 11-12 months could suggest a protective effect against the chronic low-grade inflammation related to aging and age-related diseases, mainly for females, whose treatment removed the significant differences showed between controls of 6-7 and 11-12 months.

Atherosclerosis, a chronic inflammatory process of lipid-rich lesion growth in the vascular wall that can cause life-threatening myocardial infarction (Swirski and Nahrendorf, 2013), is a typical disease of aging, to the point that increasing age is an independent risk factor for the development of atherosclerosis (Wang and Bennett, 2012). Moreover, as peripheral immune responses can trigger inflammation and exacerbation of central nervous system degeneration in several neurodegenerative diseases, an unchecked adaptive immune response in the brain can lead to neurodegeneration (Mosley *et al.*, 2012). Indeed, among age-related diseases, neurodegenerative diseases have drawn a lot of attention due to their irreversibility, lack of effective treatment, and accompanying social and economic burdens (Hung *et al.*, 2010). Also, a decrease in lymphocyte-dependent immunity can favor carcinogenesis (Miranda-Vilela *et al.*, 2013, 2014), while an undesirable modulation of the immune system invariably leads to an increase in the incidence and intensity of various diseases and ailments (Ahmad *et al.*, 2009). Given that pequi fruit contains several antioxidants and its fixed oil has been related to anti-inflammatory properties (Miranda-Vilela *et al.*, 2009c), this reinforces our suggestion that pequi may have a protective effect, as detailed above.

Consequently, our results also support the inflammation theory of aging, giving support to the argument that aging is a progressive degenerative process closely linked to inflammation (Jenny, 2012), since no significant differences in the comet assay or in the MN test related to the age groups were showed. Increases in micronuclei can occur without administration of any genotoxic agent, due to disturbance in erythropoiesis, and for this reason it is important to take into account all the toxicological and hematological findings when evaluating the genotoxicity data (FDA, 2012). In view of this, our results also support the benefits reported from pequi oil against oxidative damage to biomolecules and inflammation (Miranda-Vilela *et al.*, 2008, 2009a,b,c, 2010a, 2011a) helping in the prevention of aging-related chronic degenerative diseases.

Natural defenses against excessive generation of free radicals are based mainly on antioxidant enzymes like superoxide dismutase, catalase and glutathione peroxidase. In elderly people, these enzymes are not sufficient to prevent these oxidative processes from making the individual become more vulnerable to chronic degenerative diseases (Romano *et al.*, 2010). So, strategies that reduce age-related inflammation may improve the quality of life in older adults (Woods *et al.*, 2012).Among them, diet supplementation with antioxidant compounds, magnesium (Mg) and Zinc (Zn) in elderly rodents and humans has demonstrated decreasing oxidative rates (Hans *et al.*, 2002; Papazzo *et al.*, 2010; Eide, 2011). Like antioxidant deficiencies, magnesium (Mg) and Zinc (Zn) deficiencies have also been associated with increased oxidative stress, by increasing ROS and decreasing antioxidant enzyme expression (Mg) or by an indirect antioxidant role as an essential catalytic and structural cofactor for many enzymes (Zn) (Hans *et al.*, 2002; Eide, 2011). As oxidative stress is also implicated in the development of atherosclerosis and other chronic diseases (Hans *et al.*, 2002; Eide, 2011) related to chronic inflammation and oxidative stress, reported as a major cause of age-related diseases and cancer (Khansari *et al.*, 2009), and pequi oil is also rich in Mg and Zn, besides possessing recognized antioxidant carotenoids, its administration would be a good strategy to reduce both inflammation and oxidative stress related to aging and, consequently, related diseases.

Because diseases do not always show the usual or “classic” signs and symptoms in the elderly (Kelso, 1990), pequi oil would be a good candidate for fortifying food with a natural antioxidant while at the same time providing macro and micronutrients that have been proposed for healthy individuals, with the aim of preventing future chronic diseases like cardiovascular degeneration, diabetes and cancer (Fusco *et al.*, 2007).

In conclusion, although we did not use classic inflammation markers, the results suggest that pequi oil as a natural dietary supplementation could be a good strategy for the elderly, mainly for females, to protect against anemia, inflammation and oxidative stress related to aging, helping in the prevention of aging-related chronic degenerative diseases.
